# Evaluating county-level lung cancer incidence from environmental radiation exposure, PM_2.5_, and other exposures with regression and machine learning models

**DOI:** 10.1007/s10653-023-01820-4

**Published:** 2024-02-17

**Authors:** Heechan Lee, Heidi A. Hanson, Jeremy Logan, Dakotah Maguire, Anuj Kapadia, Shaheen Dewji, Greeshma Agasthya

**Affiliations:** 1 Nuclear and Radiological Engineering and Medical Physics Programs, George W. Woodruff School of Mechanical Engineering, Georgia Institute of Technology, 770 State Street, Atlanta, GA 30332 USA; 2 Advanced Computing for Health Sciences Section, Oak Ridge National Laboratory, 1 Bethel Valley Road, Oak Ridge, TN 37830 USA; 3 Data Engineering Group, Data and AI Section, Oak Ridge National Laboratory, 1 Bethel Valley Road, Oak Ridge, TN 37830 USA

**Keywords:** Ionizing radiation, Lung cancer, Radon, Exposome, PM_2.5_

## Abstract

Characterizing the interplay between exposures shaping the human exposome is vital for uncovering the etiology of complex diseases. For example, cancer risk is modified by a range of multifactorial external environmental exposures. Environmental, socioeconomic, and lifestyle factors all shape lung cancer risk. However, epidemiological studies of radon aimed at identifying populations at high risk for lung cancer often fail to consider multiple exposures simultaneously. For example, moderating factors, such as PM_2.5_, may affect the transport of radon progeny to lung tissue. This ecological analysis leveraged a population-level dataset from the National Cancer Institute’s Surveillance, Epidemiology, and End-Results data (2013–17) to simultaneously investigate the effect of multiple sources of low-dose radiation (gross $\gamma$ activity and indoor radon) and PM_2.5_ on lung cancer incidence rates in the USA. County-level factors (environmental, sociodemographic, lifestyle) were controlled for, and Poisson regression and random forest models were used to assess the association between radon exposure and lung and bronchus cancer incidence rates. Tree-based machine learning (ML) method perform better than traditional regression: Poisson regression: 6.29/7.13 (mean absolute percentage error, MAPE), 12.70/12.77 (root mean square error, RMSE); Poisson random forest regression: 1.22/1.16 (MAPE), 8.01/8.15 (RMSE). The effect of PM_2.5_ increased with the concentration of environmental radon, thereby confirming findings from previous studies that investigated the possible synergistic effect of radon and PM_2.5_ on health outcomes. In summary, the results demonstrated (1) a need to consider multiple environmental exposures when assessing radon exposure’s association with lung cancer risk, thereby highlighting (1) the importance of an exposomics framework and (2) that employing ML models may capture the complex interplay between environmental exposures and health, as in the case of indoor radon exposure and lung cancer incidence.

**Graphical abstract:**

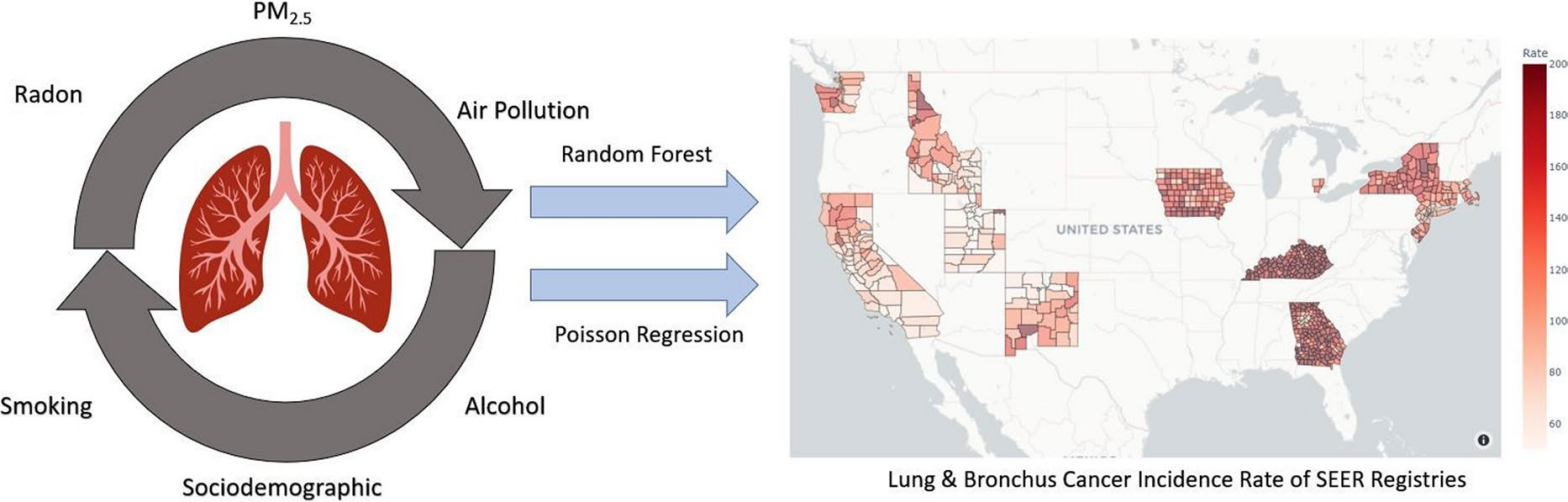

## Introduction

The concept of the human exposome was first proposed almost two decades ago as a framework to guide research that explores the etiological complexities of health and disease (Wild, 2005). Because the relationship between multifactorial exposure patterns that influence health outcomes is complex, there is a need for studies that incorporate information from multiple exposures. Approaches that include a single environmental exposure may not fully or accurately describe the risk of disease because mixing factors may alter the effects of a single exposure (Wild, 2005; Zhang et al., 2021). Alpha radiation, consisting of two protons and two neutrons, can be easily stopped by skin or paper, yet is harmful if ingested. Beta radiation, comprising electrons or positrons, can also be readily halted but poses risks to the human body when ingested. Gamma radiation, a high-energy electromagnetic wave, is produced by nuclear reactions and has strong penetrative capabilities; therefore, external exposure can cause significant harm. For example, recent studies have shown that known associations between fine particulate matter (PM_2.5_) and health are modified by gross $\beta$ activity (a measure of the counts of $\beta$ ray per unit time) (Blomberg et al., 2019; Dong et al., 2022). In the present study, we leverage statistical and ML methods to simultaneously consider the effects of background radiation levels (gamma radiation emitted from airborne radioactive particles and indoor radon gas), PM_2.5_ exposure, and other social and behavioral factors on county-level lung cancer rates.

The effects of radiation on health have long been investigated. Since the discovery of radiation and its subsequent applications in the nuclear fuel cycle, industry, security and consequence management, and nuclear medicine, the study of radiation-induced health effects on humans has been an important field of research. The evaluation of the risk from radiation exposure at high doses/high dose rates has been relatively well established through the Life Span Study of atomic bomb survivors (Seong et al., 2016; Ozasa et al., 2019; United Nations Scientific Committee on the Effects of Atomic Radiation [UNSCEAR], 2008), the Chernobyl workers study (Morton et al., 2021), and nuclear weapon fallout studies (Lyon et al., 2006; Takahashi et al., 2003). However, in determining radiation-induced outcomes, less is known about low-level or background exposures and their interactions with other environmental toxins.

### Radon Exposure and Lung Cancer

Radon is produced from the decay of uranium and is emitted from terrestrial sources and building materials. It is known that exposure to radon is affected by various factors such as housing characteristics, surficial uranium concentration, soil permeability, and even groundwater (Mose & Mushrush, 1999; Ponciano-Rodríguez et al., 2021; Przylibski et al., 2022; Smith & Field, 2007). This alpha emitter and its progenies are absorbed into the lungs, which in the process exposes the airways to radiation. Radionuclides inhaled into the lungs and bronchi cause the ionization of biological molecules, which in turn causes DNA damage and potentially cancer (Abergel et al., 2022; National Research Council [NRC], 1999; McDonald et al., 1995). In this process, most of the inhaled radon is exhaled from the lung because radon is an inert gas. However, its progenies have short half-lives that usually decay before they are removed from the lung through exhalation (Tirmarche et al., 2010). Additional complexities arise when considering the other particles and gases that the radon progeny bind to, such as PM_2.5_. The pollution particles may serve as a vector for the deposition of radon progeny into the lungs.

Epidemiological studies that link radon exposure to lung cancer risk show conflicting results (Cohen & Colditz, 1994; Kreuzer et al., 2010, 2015; Mifune et al., 1992; Yoon et al., 2016). Although there is strong evidence from studies of uranium miners (Richardson et al., 2021), studies of the broader population are less conclusive. A recent ecological study conducted in Mexico examined the relationship between indoor radon exposure and lung cancer mortality. The findings of the study suggested that higher levels of radon concentration may be linked to an increased risk of lung cancer (Ponciano-Rodríguez et al., 2021). However, this study has a limitation in that it did not control variables such as lifestyle or socioeconomic status in the model. In another study based in the USA, Cohen et al. (1994, 1995) used county-level data to investigate the association of radon exposure and lung cancer. This study showed a negative association between lung cancer risk and radon concentration, even though Cohen controlled for several confounding factors, such as smoking, socioeconomic factors, and geography (Cohen, 1995; Cohen & Colditz, 1994). There are several limiting factors within the aforementioned studies, for example, small sample sizes and challenges associated with decoupling the risk associated with radon exposure and other confounding factors (e.g., lifestyle, socioeconomic factors).

### New computational methods for population-level exposomic research

The emerging fields of data science and ML provide new opportunities for characterizing the relationship between the exposome and lung cancer by offering alternative analytical methods for modeling complex relationships between social and environmental determinants of health. Using ML methods for modeling complex relationships in epidemiology research has become increasingly prevalent (Wiemken & Kelley, 2020). This study tested the effects of low-dose radiation in single- and multi-exposure models and compared results from traditional methods and ML methods for a comprehensive look at the relationship between low-dose radiation and lung cancer rates in the USA.

## Methodology

This ecological analysis utilized county-level data to describe the relationship between two measures of low-dose radiation exposure and lung cancer rates. The following county-level factors were assembled and are summarized in Table 1: (1) environmental radiation exposures (gross gamma activity and indoor radon), (2) non-radiation environmental variables (air quality), (3) lifestyle (smoking), and (4) sociological data (demographic/socioeconomic). These variables were used to predict county-level lung/bronchus cancer risk and incidence. We tested for multicollinearity and all of the variables showed variance inflation factor (VIF) less than 5. Poisson regression and Poisson random forest (RF) regression were used to model lung cancer incidence rates in 662 counties in the USA. The MAPE (Mean Absolute Percentage Error) and RMSE (Root Mean Square Error ) from a fivefold cross-validation were compared across regression models to analyze model performance. The codes used in this analysis can be found in https://github.com/Heechan-Lee/county_radon_lung.cancer.

**Table 1:** Radiation, environmental, sociological, and cancer incidence datasets of 662 counties in the USA used in this study

Model grouping	Data	Source	Year(s)	Data description
RadNet	RadNet	EPA (EPA, n.d.)	2-year lagged averaged	Gamma count rate (interpolated & averaged), cpm
Radon	Radon zone	EPA (EPA, 1993)	1993	Zone 1: > 4 pci/L Zone 2: 2–4 pci/L Zone 3: < 2 pci/L
Radon	Radon concentration	CDC (CDC, n.d.)	2008–2017	Indoor radon tests from labs (median), pCi/L
PM_2.5_	Air quality, PM_2.5_	CDC (CDC, n.d.)	3-year lagged averaged	PM_2.5_ (averaged), µg/m^3^
Others	Air quality	CDC (CDC, n.d.)	2011	Chemicals (formaldehyde, benzene), µg/m^3^
Others	Air quality, ozone	CDC (CDC, n.d.)	3-year lagged averaged	Ozone (averaged), days 8-h average ozone concentration exceeded 0.07 ppm
Smoking	Smoking	CHR (University of Wisconsin Population Health Institute, 2022)	2015	Adult smoking rate, %
Sociodemographic	Education	SEER (NCI-DCCPS-SRP, 2022)	2008–2012	% of the population with high school education
Sociodemographic	Income	SEER (NCI-DCCPS-SRP, 2022)	2008–2012	Median family income, USD
Sociodemographic	Unemployment	SEER (NCI-DCCPS-SRP, 2022)	2008–2012	Rate, %
Sociodemographic	Urban %	SEER (NCI-DCCPS-SRP, 2022)	2010	% of the total population in urban areas
Population	Population	SEER (NCI-DCCPS-SRP, 2022)	2013–2017	Total population
Health outcomes	Lung (bronchi) cancer incidence	SEER (NCI-DCCPS-SRP, 2022)	2013–2017	Incidence count per age group and sex

### Environmental radiation data

#### Gamma count rate

**Fig. 1:**
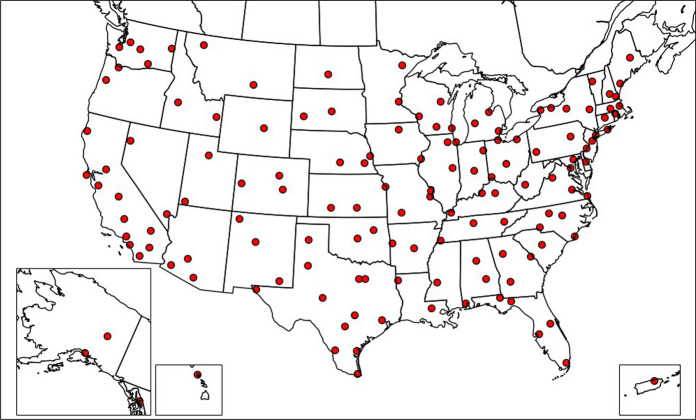
Locations of 140 counties (or equivalent) that have RadNet monitoring centers in the USA and Puerto Rico

The US Environmental Protection Agency’s (EPA’s) RadNet system monitors the gamma counts across the United States (Fraass, 2015). The first monitoring center came online in 2006, and since then the number of monitoring centers has increased to 140. (Fig. 1) Since July 2016, 80 monitoring centers also record the gamma exposure rate; however, this data is not available for the entire timeframe of the cancer incidence data, so the gamma gross count rate is used instead. Gamma gross count rates are measurements of radiation emitted from a particulate collected on an air filter—they are not a direct measure of exposure rate.

**Fig. 2:**
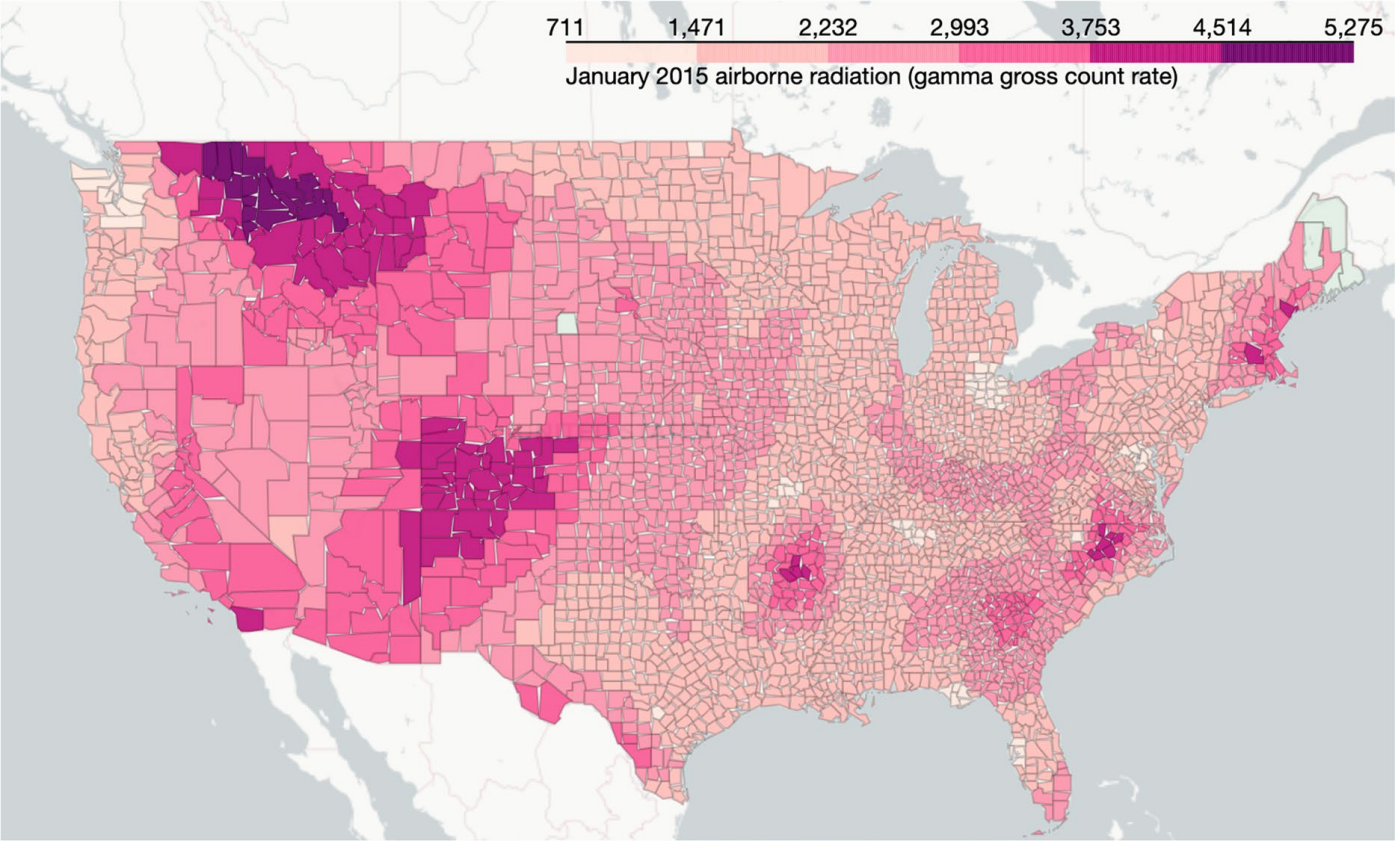
Interpolated gamma count rate (RadNet) data for the USA from data of 140 monitoring centers created with seadec function and linear interpolation

The three most prominent limitations of this dataset were as follows: (1) a high percentage of the monitoring centers were missing data from one or more months, (2) the data were limited to 140 county data points, and (3) some of the monitoring centers did not have records prior to 2013. To overcome these limitations, data were imputed by using existing alternate datasets. First, the monthly average hourly-reported gamma gross count was calculated to capture the seasonality of the data and to minimize the effect of outliers caused by local volatility. Second, the following two imputations were implemented: (1) imputing data of missing months through linear interpolation and seadec (Seasonally Decomposed Missing Value Imputation) function of R (R Core Team, 2021) from the imputeTS package(Moritz & Bartz-Beielstein, 2017) and (2) 2D linear interpolation by using the ‘griddata’ function of SciPy from Python (Virtanen et al., 2020). Linear interpolation and the seadec function outperformed interpolation with mean value as well as other methods, such as ARIMA (Autoregressive Integrated Moving Average) with Kalman filters and seasplit (Seasonally Splitted Missing Value Imputation) function of imputeTS package (Moritz & Bartz-Beielstein, 2017), for imputing the missing months. For 2D interpolation of counties without nearby or inherent geographical obstacles such as mountains or large forests, the interpolation showed less than 15% percentage error between the averaged real and predicted counts. The map of interpolated gamma count rate data is shown in Fig. 2.

We then created a summary measure of gross gamma activity for each observation year by averaging the gross gamma activity for the two years prior to the year of diagnosis (two-year lag).

#### Radon

**Fig. 3:**
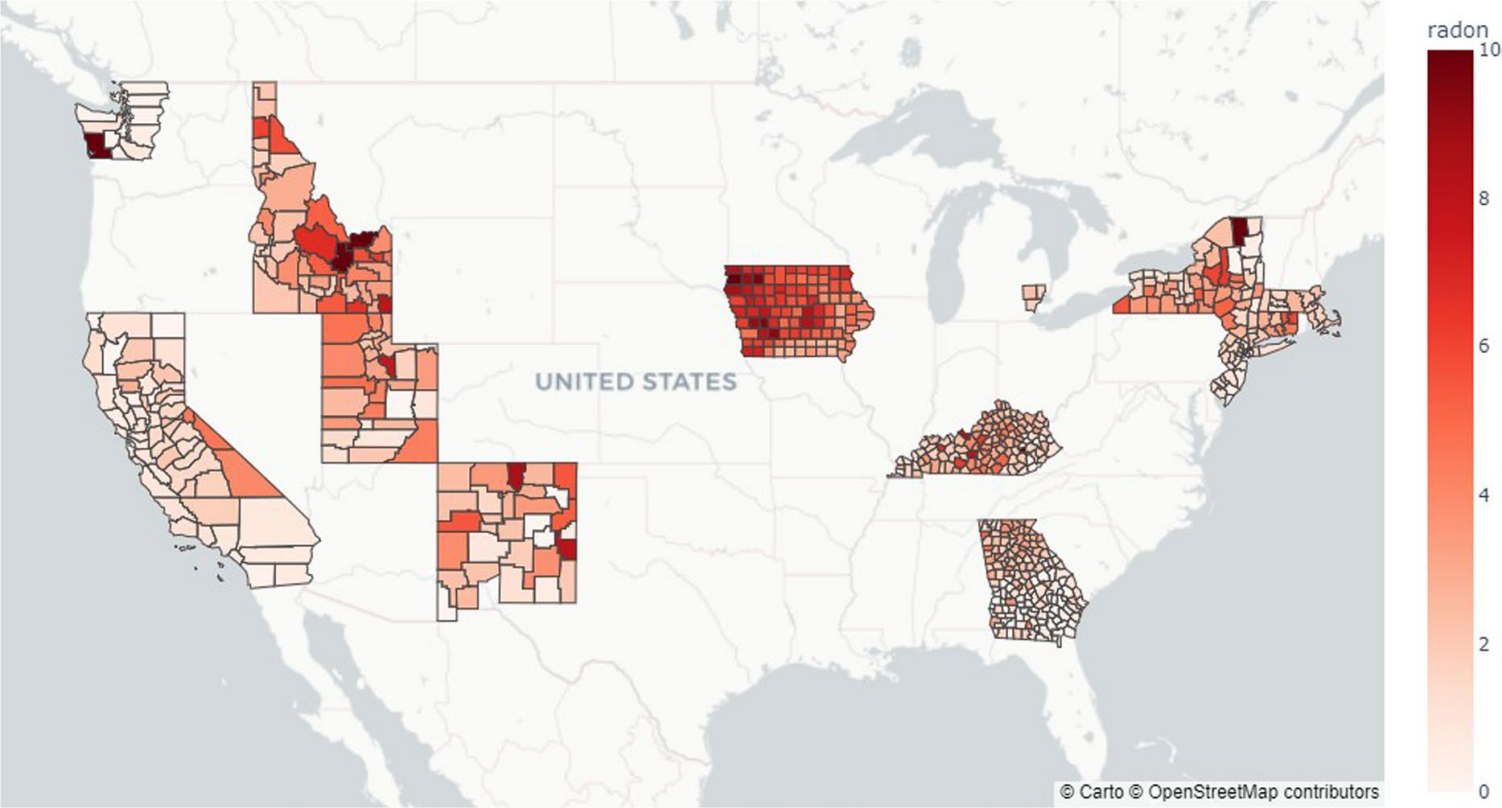
Average of yearly median indoor radon concentration for US Counties with SEER data downloaded from the US Centers for Disease Control and Prevention in pCi/L

Two sources of radon data were utilized in this study: radon zones from the US Environmental Protection Agency (1993) and the median concentration from indoor radon test kits downloaded from the US Centers for Disease Control and Prevention (CDC) database (Centers for Disease Control and Prevention, n.d.). The radon zone data classification was developed by the EPA in 1993 and classifies counties into three groups based on the potential for exposure to indoor radon: **Zone 1**, representing the highest radon concentration group of 4 pCi/L or higher; **Zone 2**, with a radon concentration of 2–4 pCi/L; and **Zone 3**, with less than 2 pCi/L (US Environmental Protection Agency, 1993). Although the classification system is almost three decades old, the classifications can reasonably be assumed as representative of the composition of soil and bedrock, which do not change significantly over this elapsed time. The CDC radon data are the results of indoor radon tests from kits deployed in residential, industrial, and educational locations across the USA from 2008 to 2017. The locations of 662 counties used in the analysis and the average of yearly median indoor radon concentrations of those counties are shown in Fig. 3.

### Non-radiation environmental data

#### Air quality

**Fig. 4:**
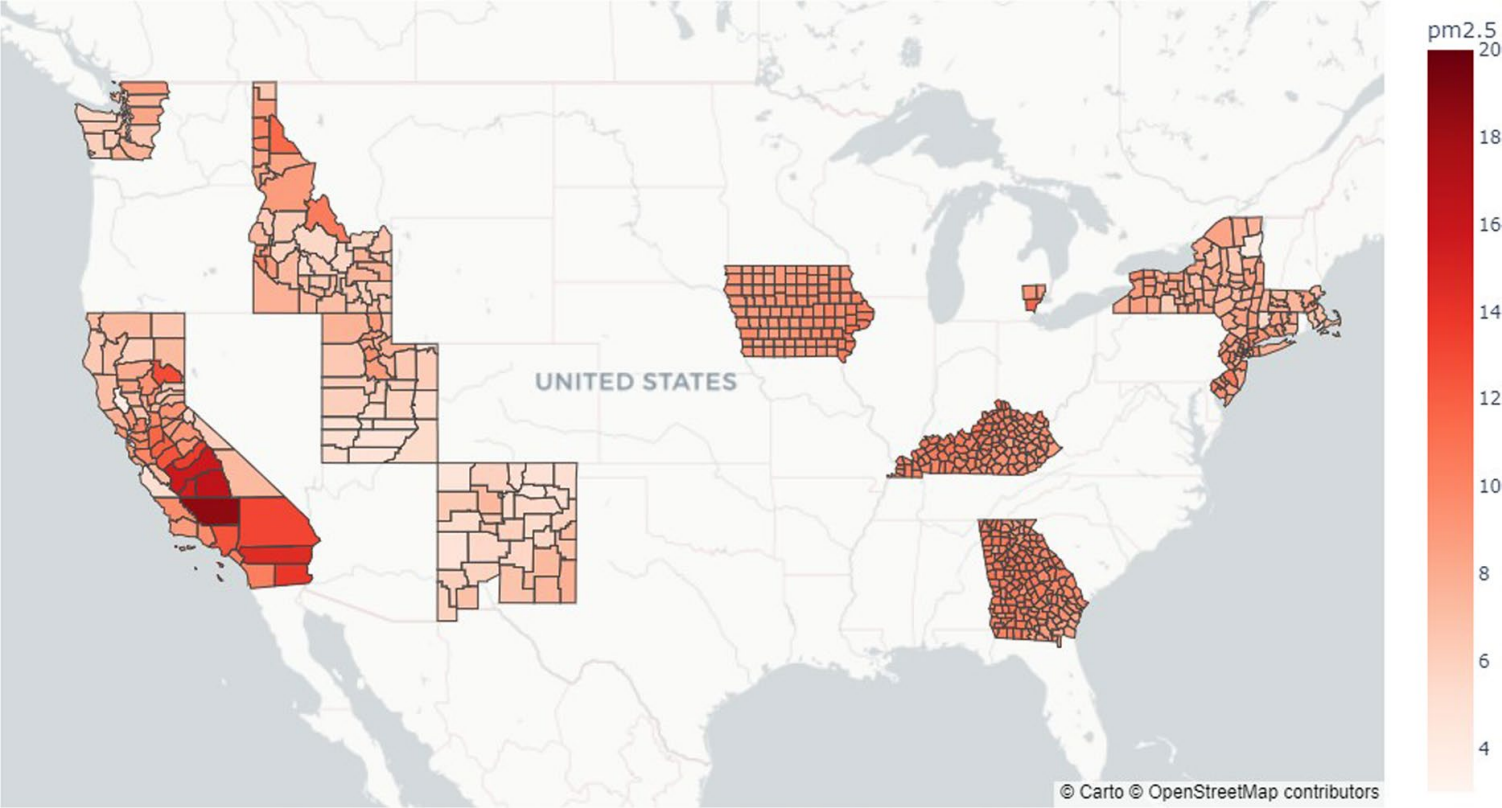
Average of PM_2.5_ concentration for US Counties with SEER cancer incidence data in µg/m^3^

Air pollution, notably particulate matter, is a known lung cancer-inducing factor (Couraud et al., 2012; Dela Cruz et al., 2011; Raaschou-Nielsen et al., 2013; Turner et al., 2011a, 2011b). Air quality-based measurements were obtained from the National Environmental Public Health Tracking Network (CDC, n.d.). This database includes various features, including toxic chemicals, ozone, and PM_2.5_. For toxic chemicals, the measurements were of an annual average concentration of 2005 and 2011. Ozone data was based on the days that the daily 8-h average ozone concentration exceeded 0.07 ppm between 2001 and 2016. The PM_2.5_ data was the average concentration of PM_2.5_ for each year between 2001 and 2016. For data on chemical concentrations, data from 2011 was extracted to best align with cancer incidence data, and the concentrations were assumed not to have changed by 2017. Among the various chemicals, formaldehyde and benzene were employed. For ozone and PM_2.5_, the average of the concentrations for the 3 years preceding the cancer incidence record was included in this analysis. The locations of 662 counties and their average PM_2.5_ concentrations are shown in Fig. 4.

### Lifestyle data

#### Tobacco smoking

**Fig. 5:**
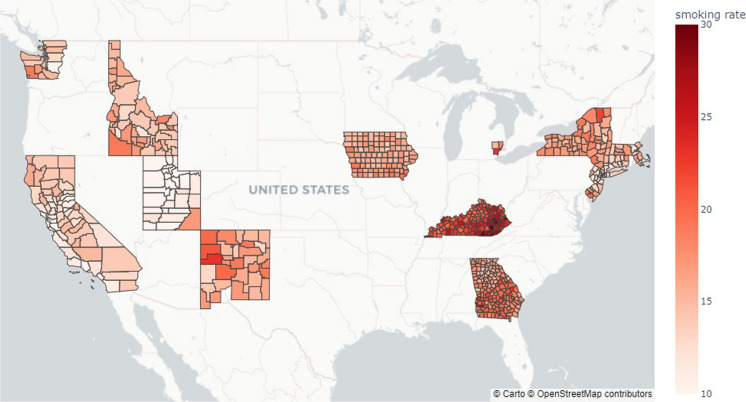
The percentage of adults in US Counties with SEER cancer incidence data who self-identified as smokers in a 2015 state-based random digit dial telephone survey of the Behavioral Risk Factor Surveillance System

Smoking has been well established as the leading lung cancer-inducing factor (de Groot et al., 2018; Dela Cruz et al., 2011). The smoking data included in this study was adapted from the Robert Wood Johnson Foundation County Health Rankings (CHR) (University of Wisconsin Population Health Institute [UWPHI], 2022; Remington et al., 2015). This dataset provides the percentage of adults who self-identified as smokers in a 2015 state-based random digit dial telephone survey of the Behavioral Risk Factor Surveillance System. The 2015 smoking rates were chosen because smoking rates are relatively constant during the period of observation, and the midpoint of interest was used as the representative year. The map of the smoking rates in 2015 are depicted in Fig. 5.

### Demographic and socioeconomic data

**Fig. 6:**
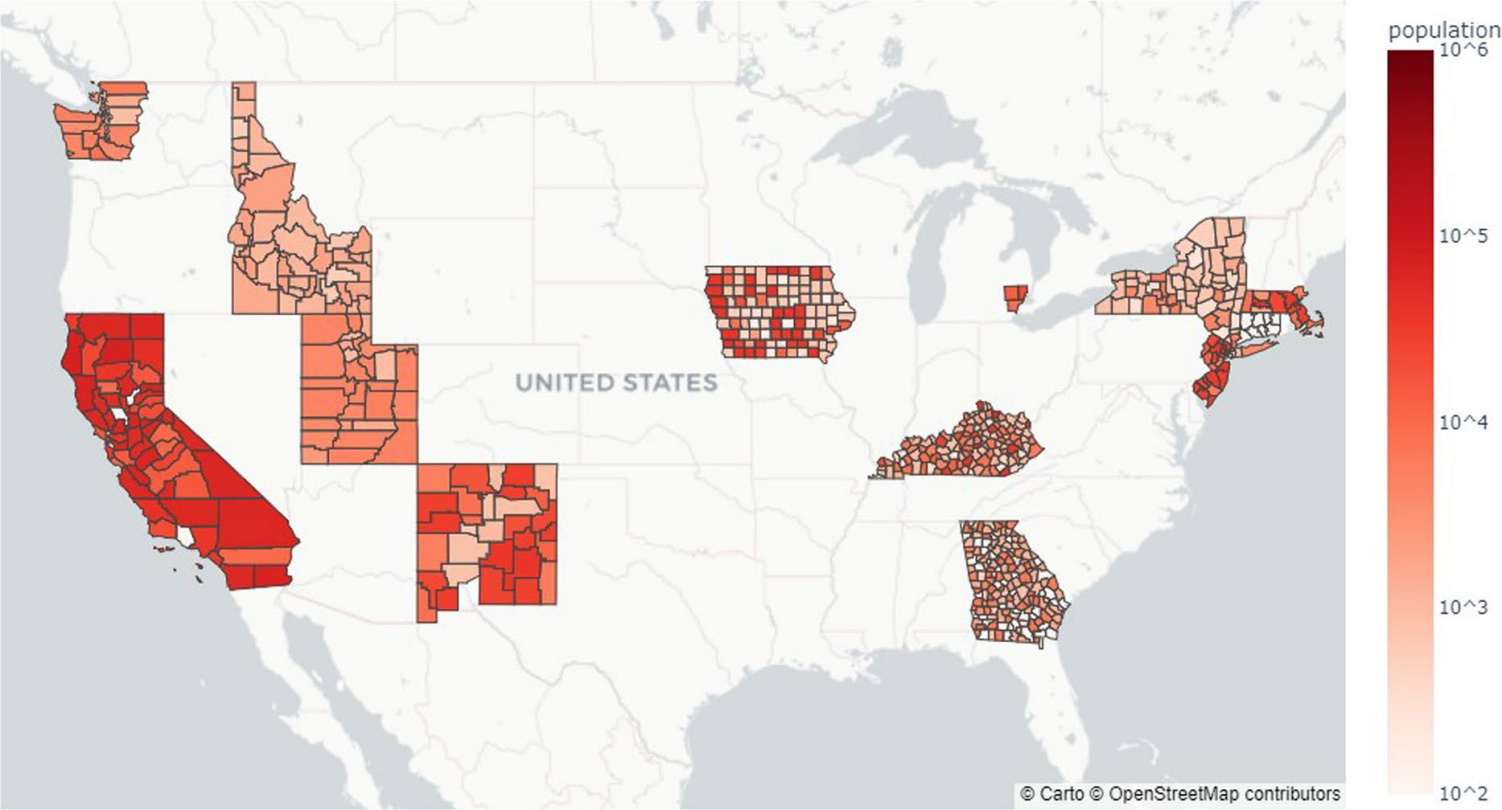
Total population in 2010 for counties with SEER cancer incidence data

Demographic and socioeconomic factors from the Surveillance, Epidemiology, and End Results (SEER) database (National Cancer Institute, DCCPS, Surveillance Research Program [NCI-DCCPS-SRP], 2022) were included in the dataset because cancer incidence is affected by various demographic and socioeconomic factors (Siegel et al., 2019). The SEER data includes education level, poverty rate, unemployment rate, rate of residence in urban areas, and divided into age cohorts separated by 5 years to reflect the age effect. Age groups range from 30–34 years old to 80–84 years old for both sexes. Age groups range from 30–34 years old to 80–84 years old for both sexes. The averages from 2008 to 2012 reported for each county were used for high school education, median family income, and unemployment data and were assumed to remain constant. The urban rate was taken from 2010 data. Total population from the 2010 US Census for the 662 countres used in the analysis are shown in Fig. 6.

### Health outcomes

#### Lung and bronchus cancer incidence rate

**Fig. 7:**
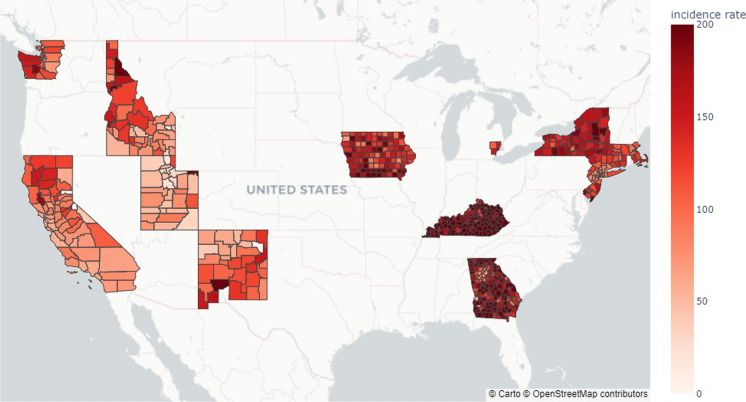
Cancer incidence rate of age-groups of interests (incidence per 100,000)

According to cancer statistics, lung and bronchus cancer cause the most cancer deaths and have the second-largest incidence across cancer types in the USA (Siegel et al., 2019). In this study, cancer incidence data employed age group and sex classifications from SEER (NCI-DCCPS-SRP, 2022). Lung and bronchus cancer incidences between 2013 and 2017 of five-year age groups, spanning from 30 to 84 years old were used in this study. This age range was carefully selected to ensure a comprehensive analysis of lung cancer incidence across adulthood, capturing variations in risk that may emerge as individuals age. Further details on age classification can be found in the SEER*Stat documentation (NCI-DCCPS-SRP, 2022). These age groups were used in this study. Figure 7 shows cancer incidence rate for the 662 counties.

### Regression models

Regression analysis was used to study the impact of various factors on health outcomes. Poisson regression, which is a count-based regression method, was utilized in this study.

The Poisson regression model used for the analysis is represented by the equation

$${\text{log}}\left({\lambda }_{i}\right)=\alpha +{\beta }_{1}{\times e}_{1}+{\beta }_{2}\times {e}_{2}+\cdots +{\beta }_{{\text{ag}}1}\times {e}_{{\text{ag}}1}+{\beta }_{{\text{ag}}2}\times {e}_{{\text{ag}}2}+\cdots +{\text{log}}({{\text{Pop}}}_{i})$$

where *λ*_*i*_ is the expected count of the outcome variables for the *i*th observation, *α* is the intercept term, *β*_*n*_ denotes the coefficient for the *n*th predictor variables *e*_*n*_ is the values of the *n*th predictor variables and the log (Pop_*i*_) is the offset term representing natural logarithm of the population for *i*th observation. For the age group variable, dummy variables were employed. *β*_ag*n*_ is the coefficient corresponding to the *n*th age group, and *e*_ag*n*_ is its associated variable. This dummy variable takes a value of 1 if the observation belongs to the *n*th age group, and 0 otherwise.

An RF approach was also employed by using rfPoisson from the R package fpechon/rfCountData (Liaw & Wiener, 2002; Pechon, 2019). The RF algorithm, which synthesizes the results from several simple trees of sequential specified questions or criteria to regress the data, can reduce the risk of overfitting on Poisson data (Pechon, 2019). The ML results were compared to Poisson regression through iterative fivefold cross-validation to evaluate the regression models. Comparisons were made with Mean Absolute Percentage Error (MAPE) (Hamner et al., 2018) and Root Mean Square Error (RMSE). In the first 5 times of fivefold cross-validation iteration, the RMSEs were computed. This was followed by a distinct 5 times of fivefold cross-validation process, in which the MAPEs and RMSEs were determined. Additionally, the variable importance measures (VIM, feature importance), which showed the importance of each factor that contributed to the regression results, was derived by using RF. VIMs were calculated using the ‘%IncLossFunction’ metric from the random forest model, which measures the percentage increase in the model's loss function when the values of that feature are randomly permuted, indicating the significance of that feature in the model's predictive performance. Incidence rate ratios (IRRs) are reported only from the Poisson regression to increase the interpretability of the results.

### MAPE

The regression results are evaluated using MAPE. This metric is scale-independent, which makes it possible to compare models across different datasets. A smaller MAPE indicates a better fitting model, where a value closer to 0 is preferred. However, it is sensitive to extreme values and positive errors. Additionally, if the actual value is close to 0, there is also a possibility that the error might be exaggerated even if the absolute error has a small value.

$${\text{MAPE}}={\text{mean}}\left(\left|\frac{g\left({x}_{t}\right)-{y}_{t}}{{y}_{t}}\right|\right)*100$$

where *g* is the regression model, and *y*_*t*_ is the target variable (de Myttenaere et al., 2016).

### RMSE

RMSE was also used for evaluating the regression results. This metric is not a scale-independent, but one of the popular statistical metrics to be used to measure the magnitude of error between predicted and observed values.

$${\text{RMSE}} = \sqrt {{\text{mean}}\left( {\left( {g\left( {x_{t} } \right) - y_{t} } \right)^{2} } \right)}$$

where *g* is the regression model, and *y*_*t*_ is the target variable.

MAPE and RMSE were calculated from a different validation process.

## Results

By developing a dataset of radiation, environmental, and sociodemographic variables that span the period of 2013–2017 (Table 1), Poisson regression and Poisson RF models were employed to model the relationship between the cancer-related factors and the lung/bronchus cancer incidence.

MAPE showed statistically significant differences when *T*-test was done between Poisson regression and Poisson RF. As the number of samples for each case is 25, degree of freedom is 48. For both males (*t*(48) = 12.86, *p *< 0.01) and females (*t*(48) = 6.40, *p *< 0.01). RMSE also showed significant differences for both males (*t*(48) = 8.85, *p *< 0.01) and females (*t*(48) = 6.57, *p* < 0.01) (Table 2).

**Table 2:** Mean absolute percentage errors (MAPEs), root mean square errors (RMSEs), and their standard deviation from the test set with Poisson regression and Poisson random forest

ML or statistical models	Male		Female	
	MAPE (SD)	RMSE (SD)	MAPE (SD)	RMSE (SD)
Poisson regression	6.29 (2.67)	12.70 (3.94)	7.13 (4.04)	12.77 (3.38)
Poisson random forest	1.22 (0.0373)	8.01 (2.54)	1.16 (0.0391)	8.15 (2.52)

Tables 3 and 4 summarize the regression results of various datasets through Poisson RF and Poisson regression. Smoking, radiation exposure, and PM_2.5_, which are thought to be related to radon exposure (Matthaios et al., 2021; Trassierra et al., 2016), and sociodemographic and behavioral factors were combined in various models. The analysis of the relationship between variables and model accuracy revealed an interesting trend in the error from the Poisson RF, as shown in Table 4. The VIM was acquired by averaging the model weights across folds with the entire dataset by using the default function of the fpechon/rfCountData package (Liaw & Wiener, 2002; Pechon, 2019). Table 5 and 6 show the VIMs of the variables analyzed with full model Poisson random forest regression including all variables, including socioeconomic variables, in the model.

**Table 3:** Mean absolute percentage errors (MAPEs), root mean square errors (RMSEs), and their standard deviation of each data set with Poisson regression

Data	Male		Female	
	MAPE (SD)	RMSE (SD)	MAPE (SD)	RMSE (SD)
RadNet+radon	8.50 (2.67)	12.22 (3.94)	7.41 (3.83)	12.52 (3.86)
RadNet+radon+smoking+PM_2.5_	7.76 (4.92)	12.75 (4.03)	20.13 (65.28)	12.66 (3.77)
RadNet+radon+smoking+PM_2.5_+others	8.79 (10.10)	12.61 (4.25)	5.95 (1.92)	12.64 (3.57)
All (Full model)	6.29 (2.67)	12.70 (3.94)	7.13 (4.04)	12.77 (3.38)

**Table 4:** Mean absolute percentage errors (MAPEs), Root mean square errors (RMSEs), and their standard deviation of each data set with Random Forest

Data	Male		Female	
	MAPE (SD)	RMSE (SD)	MAPE (SD)	RMSE (SD)
RadNet+radon	1.42 (0.0603)	7.71 (2.36)	1.27 (0.0400)	7.84 (2.60)
RadNet+radon+smoking+PM_2.5_	1.47 (0.0508)	9.21 (3.22)	1.51 (0.0323)	9.09 (3.21)
RadNet+radon+smoking+PM_2.5_+others	1.36 (0.0390)	8.61 (3.24)	1.24 (0.0305)	8.57 (2.88)
All (Full model)	1.22 (0.0373)	8.01 (2.54)	1.16 (0.0391)	8.15 (2.52)

**Table 5:** Variable importance measures (VIM) of variables from male dataset with Random Forest

Variables	VIM
	Male
Age	8.10
Smoking	0.124
Median family income	6.07*10^−2^
High school education	4.86*10^−3^
Benzene	4.84*10^−3^
Formaldehyde	3.59*10^−3^
Unemployed	7.52*10^−4^
RadNet	5.26*10^−4^
Radon	− 3.05*10^−3^
PM_2.5_	− 4.91*10^−3^
Urban	− 1.71*10^−2^
Ozone	− 2.23*10^−2^

**Table 6:** Variable importance measures (VIM) of variables from female dataset with Random Forest

Variables	VIM
	Female
Age	6.82
Formaldehyde	2.29*10^−2^
Smoking	1.61*10^−2^
Radon	9.63*10^−3^
High school education	7.11*10^−3^
Median family income	4.50*10^−3^
RadNet	4.08*10^−3^
Unemployed	1.15*10^−3^
Benzene	− 1.91*10^−3^
PM_2.5_	− 8.92*10^−3^
Ozone	− 2.89*10^−2^
Urban	− 0.158

Table 7 summarizes the IRRs analyzed with full model Poisson regression. The increased unit of the IRR is proportional to the range of each variable to make a more intuitive comparison. In both cases, smoking had the greatest effect on lung cancer incidence rates. In the case of indoor radon, the association was negative. [Male: 0.99 (0.98, 0.99), Female: 0.99 (0.98, 0.99)]. Also, Background gamma count (RadNet) [Male: 0.97 (0.97, 0.98), Female: 0.98 (0.98, 0.99)] and three-year average PM_2.5_ for female [0.99 (0.98, 1.00) *P*-value: 0.09] showed negative associations at higher concentrations, which somewhat contradicts results from previous studies (Ghazipura et al., 2019; Raaschou-Nielsen et al., 2013; Turner et al., 2011a, 2011b).

**Table 7:** Incidence rate ratios (IRRs) and 95% confidence intervals of each factor of interest with poisson regression

	IRR		Unit of measurement
	Male (95% CI)	Female (95% CI)	
Smoking	1.47 (1.45,1.48)	1.45 (1.44, 1.47)	Per 5% increase in the population
Radon	0.99 (0.98, 0.99)	0.99 (0.98, 0.99)	Per 3 pCi/L increase
RadNet	0.97 (0.97, 0.98)	0.98 (0.98, 0.99)	Per 500 cpm increase
PM_2.5_	1.02 (1.01, 1.03)	0.99 (0.98, 1.00)	Per 3 µg/m^3^ increase
Formaldehyde	1.02 (1.02, 1.02)	0.98 (0.98, 0.99)	Per 0.3 µg/m^3^ increase
Benzene	1.01 (1.00, 1.02)	1.02 (1.01, 1.02)	Per 0.3 µg/m^3^ increase

To understand the differences broken down by EPA Radon Zone, separate regression models were run for each zone using the full model Poisson regression (Table 8). In the case of Radon Zone 1, an area with high radon concentration, the effect of PM_2.5_ exposure was the greatest. Conversely, in the case of Radon Zone 3, which is an area with a low radon concentration, higher rates of PM_2.5_ were associated with lower incidence rates. The effect of smoking was consistent across all radon zones.

**Table 8:** Incidence rate ratios (IRRs) and 95% confidence intervals of PM_2.5_ and smoking by radon zone

	Radon zone 1		Radon zone 2		Radon zone 3	
	Male (95% CI)	Female (95% CI)	Male (95% CI)	Female (95% CI)	Male (95% CI)	Female (95% CI)
IRR of PM_2.5_	1.17 (1.13, 1.20)	1.13 (1.10, 1.17)	1.04 (1.03, 1.06)	1.03 (1.01, 1.05)	0.92 (0.89, 0.94)	0.94 (0.91, 0.96)
IRR of smoking	1.36 (1.31, 1.40)	1.45 (1.41, 1.50)	1.52 (1.49, 1.54)	1.55 (1.52, 1.58)	1.50 (1.47, 1.53)	1.35 (1.32, 1.37)

## Discussion

The effects of environmental exposure on health outcomes are complex. In this study, the results (Table 8) suggest that the assocation between PM_2.5_ may vary with levels of indoor radon exposure. Despite potential synergistic effects of exposure, many radiation epidemiological studies include a limited number of environmental exposure measures (Haylock et al., 2018; Richardson et al., 2015; Stanley et al., 2019; Tomasek, 2013). Belloni et al. (2020) have noted that few studies (Klebe et al., 2019; Leuraud et al., 2011) have attempted to address multifactorial exposures from environmental stressors. In the study of radiation-related disease, estimating the risk associated with radiation-related lung cancer has been a focal point in resolving the dose-risk response relationship (United Nations Scientific Committee on the Effects of Atomic Radiation [UNSCEAR], 2018). Furthermore, due to the high baseline cancer risk compared to the risk increased from low-dose radiation exposure, the population size required for detecting low-dose radiation risk with statistical significance exponentially increases as the target dose decreases (Ozasa, 2016; Ozasa et al., 2019; UNSCEAR, 2008; Valentin, 2006). To address some of the challenges, studies that use a wider range of data, such as the Million Person Study (Boice et al., 2022), are being conducted (Calabrese, 2015; Ricci & Tharmalingam, 2019; Tubiana et al., 2009; Valentin, 2008; Weber & Zanzonico, 2017). The utilization of population-level exposure variables and health outcomes data adopted in this study can serve as a valuable resource for future research. Population-level data offers an advantage in the adoption of multiple variables and the analysis of diverse health outcomes. Furthermore, ML techniques are particularly well suited to model the complex relationships that exist between environmental exposure and health outcomes. By leveraging ML, it is possible to capture the complex interplay between environmental exposures and health, thereby offering a promising avenue for future research in this field.

The results suggest that PM_2.5_ should be included in future analysis of radon-induced lung cancer incidence, as there may be an interaction with radon exposure. The observed patterns, where changes in radon concentration result in significant differences (*p *< 0.001 for all cases) in the effects of PM_2.5_, corroborate findings from other research that explores the combined impacts of PM_2.5_ and radon exposure (Dlugosz-Lisiecka, 2016). PM_2.5_ or other particulate matter could be one of the possible transport mechanisms that allow radon gas to permeate lung tissue. This is further supported by two experimental studies that assess the speciation of PM_2.5_ particles in the presence of radon progeny. The first study shows that the alpha activity of PM_2.5_ tends to increase as the concentration of radon increases (Matthaios et al., 2021). The second study shows that in a radon chamber, the presence of particulate matter will increase the attached fraction of radon progeny, thereby implying that the radiation exposure from particulate matter will increase (Trassierra et al., 2016). PM_2.5_ and radon seem to have synergistic effects and are thought to affect various health outcomes, including incidences of lung cancer. Given the possible synergistic effect between PM_2.5_ and radon, future epidemiological studies should investigate this further.

This study harnessed ML to consider the non-linear effects of radon exposure within the context of other environmental factors. The results of decreased errors from ML models show that ML is effective at analyzing complex relationships in environmental exposure studies and should be considered in future studies that investigate the relationship between radon exposure and cancer outcomes. One limitation of current ML is the lack of variety in ML algorithm packages that can be applied to count data. However, it is believed that these problems will naturally be resolved as ML develops and becomes more widely used in regression analysis.

Large-scale data can be challenging when conducting analysis attributable to individual characteristics, for example they are limited in their ability to reflect the interaction of environmental and genomic factors, which is important in the exposome approach (Zhang et al., 2021). Furthermore, individual history of exposure information which is similarly essential to exposome analysis is difficult to reflect in the analysis (Zhang et al., 2021). Thus, population-level studies of incidence rates, such as this one, are susceptible to the ecological fallacy. This limits the ability to establish causal relationships between variables and health outcomes. Despite these limitations, population-level studies can still provide valuable reference points for guiding individual-level studies.

The World Health Organization (2009) reported that radon is the second major contributor to lung cancer incidence. Also, a study by Turner et al. (2011a, 2011b), which analyzed county-level radon concentrations and residents' lung cancer risk similar to this study, showed a positive association between residential radon and lung cancer risk. However, our results showed that there was negative association between radon and lung cancer incidence rates [IRR of male: 0.99 (0.98, 0.99), IRR of female: 0.99 (0.98, 0.99)]. There are several reasons our findings may differ from occupational cohort studies that show there is a strong association in occupational studies where individuals are exposed to high levels radon (Kreuzer et al., 2015; Leuraud et al., 2011; Richardson et al., 2021, 2022). First, as mentioned above, this study may suffer from ecological fallacy. Second, indoor radon exposure risk is measured at the county level and radon exposure varies widely across counties (Li et al., 2021). Third, the effect sizes at low levels of exposure are likely small—making the signal difficult to detect in an ecological analysis. Our results of study which investigated the association between residential radon exposure to lung cancer is difficult distinguished are more aligned with results from recently published residential exposure and lung cancer-based study (Li et al., 2020). The study on residential radon exposure and lung cancer risk in Connecticut and Utah (Sandler et al., 2006) could not provide evidence of an increased risk of lung cancer at the exposure levels observed. Unlike minor studies, the residential radon exposure is so low that statistically significant results are difficult to obtain.

Furthermore, the difference in findings across studies may arise from discrepancies between individual-level and population-level approaches in their methodologies and analysis. Also, regarding the interaction between smoking and radon, the results were different from the previous studies. According to BEIR VI, a comprehensive analysis of the relationship between smoking habits, radon exposure, and lung cancer risk of uranium miners from several studies showed a submultiplicative effect, which means that the risk in the population exposed to both smoking and radon is greater than the sum of the individual risks expected from either smoking or radon exposure and less than the product (NRC, 1999). The results of a case-control study in Spain after BEIR VI indicated that there is a strong synergistic effect between smoking and radon exposure, and the case-control miner study showed evidence of submultiplicative interaction between radon and smoking (Barros-Dios et al., 2012; Leuraud et al., 2011). However, the association between smoking and radon concentration did not appear to be significant in the results presented herein. These inconsistent results again may be attributed to certain limitations in this study, including terse measurement of radon concentrations. Using the median data could prevent the effects of outliers, but it will have errors from the insufficient number of tests. This problem could skew the results toward non-significant associations or even contradict established knowledge.

Possible confounding factors that were not properly reflected are that the level of stress that people experience, and the quality of medical care will vary considerably by county or state despite some socioeconomic factors being included. This may also explain the opposite trend in this analysis vs. the previously known results of PM_2.5_ and lung cancer incidence. These problems could be mitigated if the research is conducted on specific regions with very high-resolution data, or by improving our measures of radon concentrations. Another limitation of this study is the lack of residential history data, which made it impossible to create a model that adequately considers different exposures across a life span and the associated latency periods. Other lung cancer models have considered the incubation period of 5 years (National Research Council [NRC], 2006; UNSCEAR, 2008; Valentin, 2008). Future studies should use residential history to assess the effects of indoor radon exposure across a life span.

If future studies address these limitations, then the combination of highly accurate ML techniques and the advantages and applicability in radiation epidemiology of population-level data could be harnessed for more diverse health outcome analysis. This may also provide valuable insights into the interplay between variables.

## Conclusion

Traditional statistical methods and ML models can be used in parallel to fully understand the complex relationship between environmental exposures and health. To investigate the applicability of multivariable and ML methods in environmental exposure studies, county-level lung/bronchus cancer risk was assessed with various exposures (airborne gamma counts, radon concentration, air quality), lifestyle (smoking), and socioeconomic factors through Poisson regression and Poisson RF regression. The study found that the risk of lung cancer from PM_2.5_ varied by radon concentration with larger effect sizes in areas with high indoor radon exposure. In summary, the results of this study demonstrate how (1) including multiple environmental exposures has advantages over single exposure studies when the relationship between the environment and lung cancer risk is considered, thereby making an exposomics framework an important consideration, and (2) employing ML models enhances the utility of analysis in identifying complex relationships, as in the case of environmental radiation exposure and lung cancer incidence. Consequently, this study proposes a new paradigm for studying environmental radiation combined with other environmental exposures.
